# Confounding Factors in the Transcriptome Analysis of an *In-Vivo* Exposure Experiment

**DOI:** 10.1371/journal.pone.0145252

**Published:** 2016-01-20

**Authors:** Oskar Bruning, Wendy Rodenburg, Paul F. K. Wackers, Conny van Oostrom, Martijs J. Jonker, Rob J. Dekker, Han Rauwerda, Wim A. Ensink, Annemieke de Vries, Timo M. Breit

**Affiliations:** 1 RNA Biology & Applied Bioinformatics research group, Swammerdam Institute for Life Sciences, Faculty of Science, University of Amsterdam, Amsterdam, the Netherlands; 2 Centre for Health Protection (GZB), National Institute of Public Health and the Environment (RIVM), Bilthoven, the Netherlands; Queen's University Belfast, UNITED KINGDOM

## Abstract

**Confounding factors:**

In transcriptomics experimentation, confounding factors frequently exist alongside the intended experimental factors and can severely influence the outcome of a transcriptome analysis. Confounding factors are regularly discussed in methodological literature, but their actual, practical impact on the outcome and interpretation of transcriptomics experiments is, to our knowledge, not documented. For instance, *in-vivo* experimental factors; like Individual, Sample-Composition and Time-of-Day are potentially formidable confounding factors. To study these confounding factors, we designed an extensive *in-vivo* transcriptome experiment (n = 264) with UVR exposure of murine skin containing six consecutive samples from each individual mouse (n = 64).

**Analysis Approach:**

Evaluation of the confounding factors: Sample-Composition, Time-of-Day, Handling-Stress, and Individual-Mouse resulted in the identification of many genes that were affected by them. These genes sometimes showed over 30-fold expression differences. The most prominent confounding factor was Sample-Composition caused by mouse-dependent skin composition differences, sampling variation and/or influx/efflux of mobile cells. Although we can only evaluate these effects for known cell type specifically expressed genes in our complex heterogeneous samples, it is clear that the observed variations also affect the cumulative expression levels of many other non-cell-type-specific genes.

**ANOVA:**

ANOVA analysis can only attempt to neutralize the effects of the well-defined confounding factors, such as Individual*-*Mouse, on the experimental factors UV-Dose and Recovery-Time. Also, by definition, ANOVA only yields reproducible gene-expression differences, but we found that these differences were very small compared to the fold changes induced by the confounding factors, questioning the biological relevance of these ANOVA-detected differences. Furthermore, it turned out that many of the differentially expressed genes found by ANOVA were also present in the gene clusters associated with the confounding factors.

**Conclusion:**

Hence our overall conclusion is that confounding factors have a major impact on the outcome of *in-vivo* transcriptomics experiments. Thus the set-up, analysis, and interpretation of such experiments should be approached with the utmost prudence.

## Introduction

About two decades ago, the arrival of microarray technology for genomics and transcriptomics plus similar genome-wide techniques for proteomics and metabolomics led to a number of major developments in experiment design, laboratory execution, and data analysis [1–12]. Over the years these techniques matured by enhancing the detection levels, reducing the technical noise, improving the bioinformatics analyses, and so on, resulting in greatly improved detectors compared to previous techniques. This process is still ongoing as can be seen by the developments in the latest omics innovation: third-generation sequencing [13–16].

In contrast to the spectacularly improving omics technologies, the mechanistic knowledge of biological systems gained by employing these technologies is relatively slowly progressing. Although microarray technology and next-generation sequencing for instance, have proven their potential in biomarker applications and genome-wide screening approaches, regular transcriptome studies into unravelling gene-expression pathways and networks, however, often result in limited new insights. This has been puzzling life sciences researchers ever since these technologies became available [17–20]. What we have become to appreciate is that gene-expression involves highly complex and multilevel networks, which are extremely difficult to unravel.

Another reason for the omics struggle, as observed earlier by us and others, is that we might not use the appropriate omics experimental designs to study these complex systems [19,21,22]. For instance, perturbations with a too high intensity might lead to a generic stress reaction, rather than a response specific to the perturbation. Also, an appropriate number of replicates are needed in order to have enough statistical power to methodically analyze omics experiments involving tens of thousands of genes [23–25]. As the issues are well known, much effort has been invested to improve them. Despite all enhancements, the knowledge gain by omics experiments is still below expectation. This might also be due to elusive experimental factors that confuse the analysis of the experimental results. These so-called confounding factors can include an endless array of issues, such as the effects of circadian rhythm during a day or fluctuating oxygen levels during cell culturing [26].

Although most biologists are typically quite aware of such factors, they tend to accept and/or ignore them as an inevitable fact-of-life in biological *in-vivo as well as in-vivo* experimentation. It is frequently argued that the effects of such factors result in “random noise”. However, if these factors interact with the biological system studied and are confounding i.e. biasing the results, then they will have a significant impact on the analysis and interpretation of the results. As confounding factors are an integral part of any biological system, it can safely be asserted that every experiment involving living cells will suffer from several such (unknown) confounding factors.

The concept of confounding factors is already well established in statistics and methodology and there are many methods to counter their effects, such as, for instance, adjusting the experiment design and by statistical hypothesis testing using Analysis Of Variance (ANOVA) [27–29]. There might be confounding factors that cannot be totally separated from the experimental factors, leading to a form of partial confounding. In addition, in order to be able to model such partial confounding factors, for instance as nuisance factors, they obviously have to be defined. We however hypothesize that in transcriptome experiments confounding factors can be present that cannot be defined a-priori.

Thus, we set out to evaluate the effects of some potential confounding factors in a large-scale *in-vivo* transcriptome experiment of over 260 samples involving UVR exposed murine skin. For this, we used an optimized experiment design with respect to the technology and UV-Dose range, as established earlier [30]. In order to allow for evaluation of mouse-dependent confounding factors, the experiment design included up to six samples per individual mouse, which makes it quite unique.

We identified several confounding factors in our study: Sample-Composition, Time-of-Day, Handling-Stress, and Individual-Mouse. We evaluated the origin of these factors and their influence on the results of the experiment. It became clear that these confounding factors significantly affected the measured expression of many genes, also genes that were identified as differentially expressed in the intended experimental contrasts. We will discuss the consequences of these confounding factors on the outcome of this experiment and transcriptomics experimentation in general. As the severe effects of the confounding factors that we have found here are not exclusive to this study or *in-vivo* experimentation, our study should be read as a cautionary tale for all biological researchers using transcriptomics experiments in their research. Knowledge and understanding of confounding factors will at least put transcriptomics results in a clearer biological perspective, or better yet, force the experimenter to take a hard look at the design for transcriptomics experimentation.

## Material & Methods

### Ethics Statement

This study was agreed upon by the Animal Experimentation Ethical Committee (AEEC) of the RIVM in Bilthoven, the Netherlands under permit number 201200128. Animal handling in this study was carried out in accordance with relevant Dutch national legislation, including the 1997 Dutch Act on Animal Experimentation.

Biopsies were taken under Isoflurane anesthesia, at the end of the study animals were euthanized by cervical dislocation and all efforts were made to minimize suffering.

### *In-vivo* UVR exposure experiment

Generation of the mouse model has been described previously [31]. All mice at least 10 times backcrossed in SKH hairless strain, and sexed at 3 weeks of age. During the whole experiment wild-type (WT; *Trp53*+/+), *Trp53*72R/72R, and *Trp53*72P/72P mice were maintained under specific pathogen-free conditions with a target ambient temperature of 21°C, humidity of 40–70% and with a 12/12 h light/dark cycle. Mice were single-housed in standard Macrolon type II cages and were fed SRMA diet (hope farm) and water ad libitum. Supposed males of 7–10 weeks of age were UVB radiation exposed at 2 different doses (90, 540 J/m^2^) in a chamber containing Phillips TL12 lamps. Control mice were mock treated. At various time points after treatment (0, 7.5, 9, 10.5, 12, 13.5 hr. for the 90 J/m^2^ dose and 0, 1, 2, 3, 4, 5 hr. for the 540 J/m^2^ dose) both treated and untreated mice were anaesthetized by isoflurane. Subsequently, 1.5 mm biopsies were sampled from the center dorsal skin by punching a half moon shape on folded skin using sterile biopsy punches with plunger system (Kai Europe gmbh Solingen, Germany). Biopsy wounds were closed using sterile Histoacryl-tissue-adhesive, wounds were monitored until the end of the experiment. Previously, a similar experimental setup also showed no infection signs such as signs for infection such as redness or swelling appeared under the conditions used [30]. At 48 hr. after treatment all mice were euthanized by cervical dislocation after biopsies were taken. Biopsies were immediately snap frozen in liquid nitrogen and stored at -80°C until further processing. Total-RNA was isolated as previously described in [13]. In short, frozen skin punch biopsies were pulverized in liquid nitrogen and immediately transferred to 0.3 ml Qiazol reagent (Qiagen). RNA was extracted according to the manufacturer’s instructions with the addition of Phase-Lock Gel Heavy (5 Prime) to obtain a better phase separation. Further clean-up of the RNA fractions was performed using the RNeasy Minelute Cleanup Kit (Qiagen). RNA yield was measured on a Nanodrop ND-1000 (Thermo Fisher Scientific) and RNA integrity was evaluated using High Sensitivity R6K ScreenTapes on a 2200 TapeStation instrument (Agilent Technologies). RINe values were at least 6, with the exception of two samples (RINe 4.4 and 5.8). An overview of all samples is shown in Table 1 plus S1 Table.

**Table 1:** Experiment set up for WT mice.

			Recovery Time (hours)									
			Early					Late				
Trp53-Genotype	UV-Dose(J/m2)	Individual-Mouse (#)	0	1	2	3	4	5	7.5	9	10.5	12
WT	540 (high)	32	1	1	1	1	1	1				
		38	1	1	1	1	1	1				
		44	1	1	1	1	1	1				
		50	1	1	1	1	1	1				
	None	31	1	1	1	1	1	1				
		37	1	1	1	1	1	1				
		43	1	1	1	1	1	1				
		55	1	1	1	1	1	1				
	90 (low)	8	1						1	1	1	1
		14	1						1	1	1	1
		20	1						1	1	1	1
		26	1						1	1	1	1
	None	1	1						1	1	1	1
		13	1						1	1	1	1
		19^a^	1						1	1	1	1
		25	1						1	1	1	1

^a^ Data from Mouse 19 was removed before data analysis (cf. text)

### Microarrays with custom Mouse Agilent platform

Gene expression levels of the mouse samples were analyzed with a 4x180k *Mus musculus* microarray (Custom design GEO Platform accession number GPL19390) containing 24,203 genes based on NCBI-GeneID. All procedures were performed according to manufacturer’s instructions. Briefly, for each test sample, 100 ng total-RNA was combined with Spike A and subsequently amplified and labeled using the Quick Amp Labeling Kit (Agilent Technologies). For use as common reference, an equimolar pool of all test samples was made and amplified as described above with the exception that Spike B was included and Cy5 was used for labeling. Amplified cyanine-dye labeled antisense RNA was purified using the RNeasy MinElute Cleanup Kit (Qiagen) and yield and dye incorporation were evaluated using a NanoDrop ND-1000. Labeled test samples and common reference samples were combined and hybridized to the Agilent custom microarrays (Agilent Technologies). After washing, the arrays were scanned with an Agilent DNA microarray scanner (G2565CA, Agilent Technologies) and data was extracted with Feature Extraction software v10.7.3.1 (Agilent Technologies).

The array data have been deposited in NCBI's Gene Expression Omnibus (GEO) and is accessible through GEO Series accession numbers GSE63044.

### Microarray data processing

The quality of the microarray data was assessed via multiple quality-control checks, i.e. visual inspection of the scans, testing against criteria for foreground and background signals, testing for consistent performance of the labelling dyes, checking for spatial effects through pseudo-color plots, and inspection of pre- and post-normalized data with box plots, ratio-intensity (RI) plots and PCA plots. All arrays passed the minimal criteria for quality assessment of the microarray data and were used in the analyses.

Handling, analysis and visualization of all data was performed in R (http://cran.r-project.org/) using the Bioconductor (http://www.bioconductor.org/) packages limma and maanova.

Log2 transformed data was normalized within-array using LOESS on an MA-plot of the Cy3 test sample data vs. the corresponding Cy5 reference sample data. Subsequently, the robust multi-array average (RMA) algorithm was performed only on the normalized Cy3-sample data for between-array normalization through summarization of the intensity values of the probes in a NCBI-GeneID probe set.

### Data analysis

Before any data analysis, the quality of the experiment data was evaluated. For this we looked at genotype, gender, rRNA yield, and mRNA yield (S1 Text).

To visualize various effects of confounding factors on the complete transcriptome Principal Components Analysis (PCA) plots were made in R using the RMA normalized data. For further analyses we presumed that confounding factors are relevant if they cause clusters of genes to have high expression values in clusters of samples. As a consequence, confounding with the experimental factor “UV-Dose” can lead to wrong biological conclusions.

To investigate this, the top 5,000 of most variable genes over all untreated WT samples were selected, and clustered using Ward’s method (with the average value used as ordering function). The resulting heatmap was studied for clusters of genes (known to be) related to the confounding factors “Sample-Composition” and “Time-of-Day”. Gene clusters related to Sample-Composition were checked for co-expression and tissue specific expression in an independent data set: the BioGPS data base (http://biogps.org/).

The confounding factor Time-of-Day was further characterized by screening for those genes that display a high fold change (log2(FC)>|1|) between any two time points in each individual mouse (although the time points might differ), as these genes are potentially under circadian control in this experiment. This gene list, related to the confounding factor “Time-of-Day”, was partly confounded to “Sample-Composition”.

The effect of the confounding factor “Handling-Stress and Biopsy-Stress” was quantified applying a statistical analysis for differential gene expression using a mixed linear model with coefficients for biopsy ranking numbers (fixed) and Mouse (random), thus treating biopsy as experimental variable. The allocation of samples to the biopsy ranking numbers (B1, B2, B3 and B4) is explained in the “Results & Discussion” section. A contrast analysis was used to test differential expression between B1 vs B2, B1 vs B3 and B1 vs B4, for hypothesis testing a permutation based Fs test was used [32], and the resulting P-values were corrected for false discoveries according to Storey and Tibshirani [33]. Differentially-expressed genes (DEGs) were identified by applying a significance cut-off (FDR corrected P-value < = 0.05). The DEGs from the different contrasts were combined and ranked from low to high variance, which was calculated as described previously.

The effect of the confounding factor “Individual-Mouse” was quantified by applying statistical tests for differential gene expression with and without “Individual-Mouse” as random covariate, and analyzing the results. The statistical tests were performed as described above, using a model with a group-means parameterization based on the time point–UV-Dose combinations. The model coefficients quantifying the Individual-Mouse effects were used to select the genes: the absolute coefficient values had to be higher than 0.5 and the genes should not be found in any of the other gene sets.

To learn more about the effects of the different subsets of genes that were defined in the preceding paragraphs, boxplots were created of the different elements of the ANOVA model used for the Individual-Mouse effect. A 2x2 Self-Organizing Maps (SOMs) analysis was performed on all gene sets associated with all confounding factors, in order to compare the variability in gene expression within each mouse with the variability across all mice.

To determine the impact of the overlap between the DEGs and the genes in the clusters and groups an over-representation analysis was performed. Here the DEGs, gene clusters/groups and the array background were compared using the hypergeometric test.

## Results & Discussion

Confounding factors can severely hamper proper transcriptome analysis, especially since biologists often design complex multi-variable experiments. The transcriptomics experiment we use here to research confounding factors is no exception, as it contains several variant genomes, a perturbation and recovery time as experimental variables.

In this study, we obtained six consecutive samples from each individual mouse [30,34], which differs from most *in-vivo* murine transcriptome studies, where sampling restrictions force the use of individual mice per time point and often also per permutation. Our exceptional experiment design provided us with a unique opportunity to examine several confounding factors that may have an impact on the mouse transcriptome. This would normally be impossible due to the considerable differences between individual mice.

Besides the intended static experimental factor (*Trp53*-Genotype) and intended variable factors (UV-Dose and Recovery-Time), there are several “uncontrollable” experimental factors we foresaw as confounding factors; Individual-Mouse, Sample-Composition, Handling- & Biopsy-Stress, Time-of-Day (Table 2). Even though, the experiment was meticulously set-up, we will investigate the impact of these confounding factors and their effect on the analysis and interpretation of the experimental transcriptome data.

**Table 2:** Experimental factors affecting the transcriptome.

Factor	Type			*Figure*
UV-Dose	Intentional	Controllable	Variable	*3A*
Recovery-Time	Intentional	Controllable	Variable	*3B*,*C*
Trp53-Genotype	Intentional	Controllable	Static	*3D*
Sample-Composition	Confounding	Uncontrollable	Dynamic	*3B*, *4*, *5*
Time-of-Day	Confounding	Uncontrollable	Dynamic	*3B*, *7*, *8*
Handling-Stress	Confounding	Uncontrollable	Dynamic	*3E*, *9*
Biopsy-Stress				
Individual-Mouse	Confounding	Uncontrollable	Dynamic	*3F*, *10*

### Experimental set-up

The underlying biological research aim of the experiment was to investigate the cellular responses upon UVR induced DNA damage. For this, an *in-vivo* experiment was performed with UVB radiation exposed skin of nude, male mice, with and without human-derived *Trp53* variants in their genome. This resulted in an extensive experiment design as showed in Table 1 (and S1 Table). Essentially, four replicate mice were used for each UV-Dose. Each mouse was sampled five to six times at different recovery time points after UV-pulse exposure, which resulted in paired samples. In total 132 treated and 132 untreated samples were taken from 64 male mice.

### Global impression of transcriptome variation

**Fig 1:**
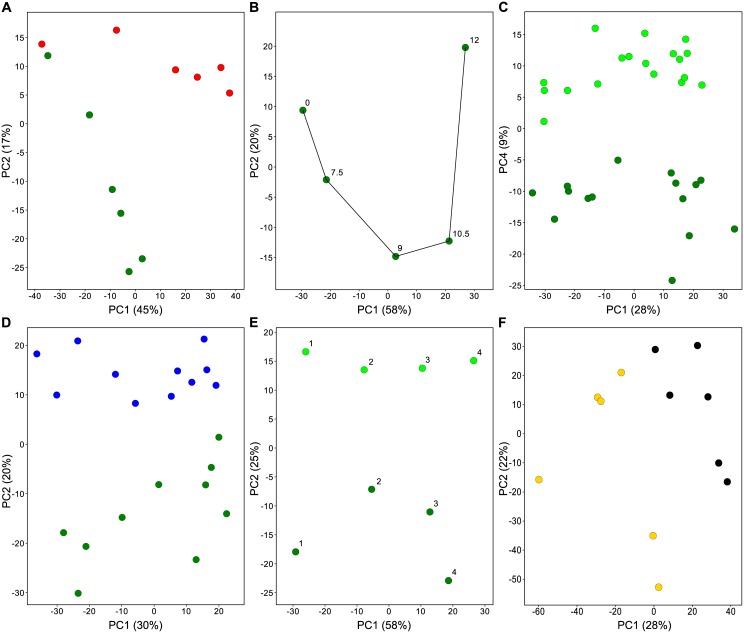
Variation caused by confounding factors. Selected samples to exemplify the influence of confounding factors (Table 2) derived from the PCA analysis of the complete experiment. Sample means are used except for D and E. A, UV-Dose (green = untreated, red = high dose); B, Recovery-Time (hours) ≈ Time-of-Day; C, Recovery-Time (light green = early, green = late); D, Trp53-Genotype (green = WT, blue = *Trp53*-72P mutant); E, Handling- & Biopsy-Stress (light green = Early recovery, green = Late recovery); and F, Individual-Mouse (yellow = #31, black = #55).

As stated earlier, experimental factors can be (un-)intentional, (un-)controllable, and static/variable/dynamic (Table 2). The unintentional and uncontrollable factors are potential confounding factors of importance. However, as the effect of confounding factors can, by definition, not be distinguished from each intended experimental factor, their effect can also not always be separated from each other. For instance, the effect of the factors “Sample-Composition” and “Time-of-Day” is also confounded. This is also the case for factors “Handling-Stress” and “Biopsy-Stress”. Plus there are fuzzy confounding factors, such as “Individual-Mouse” that are an accumulation of many possibly confounding factors like genetic constitution, health, behavior, and so on. To get an impression of the impact of our foreseen experiment factors on the variation in the experiment, we performed a PCA analysis in which we calculated the principal components from the gene expression values (Fig 1). In the PCA plots, it was possible to distinguish groups of samples as defined by intended, but also non-intended experimental (confounding) factors. This shows that these confounding factors have a detectable impact on the variation in the experiment. We evaluated the mentioned confounding factors to estimate their disturbing effect on the transcriptome analyses of the intended experimental factors, UV-Dose and Trp53-Genotype.

### Potential confounding factor: Sample-Composition

The observed differences in the total-RNA yield of the samples (S1 Fig), raised suspicion that the composition of the biopsy samples might be quite variable. The biopsies in this study are obtained by stretching and folding the skin of a mouse and punching out a double half circle [35]. This is not a fully controlled procedure; hence we anticipated that variability in sample composition could be a confounding factor of significance. Any biased variation in cell type composition between samples will result in measurements of RNA levels that could be misinterpreted for differential gene expression [36–39]. This is especially true for genes that are specific for one cell type [36,37]. Fluctuations in the contributing fraction of a particular cell type in a sample will translate immediately into a signal for a cell type-specific gene that mimics differential gene expression. Although on an individual gene level it is impossible to distinguish whether RNA level differences are caused by differences in sample composition or differential gene expression, on a gene set level this distinction can be made. Because, if the RNA level difference is caused by changing the percentage of certain cells in a sample, the RNA levels of all cell type-specific genes will change in an identical way over many samples. In this way we can identify genes and thus cell types with differential RNA levels caused by differences in sample composition.

**Fig 2:**
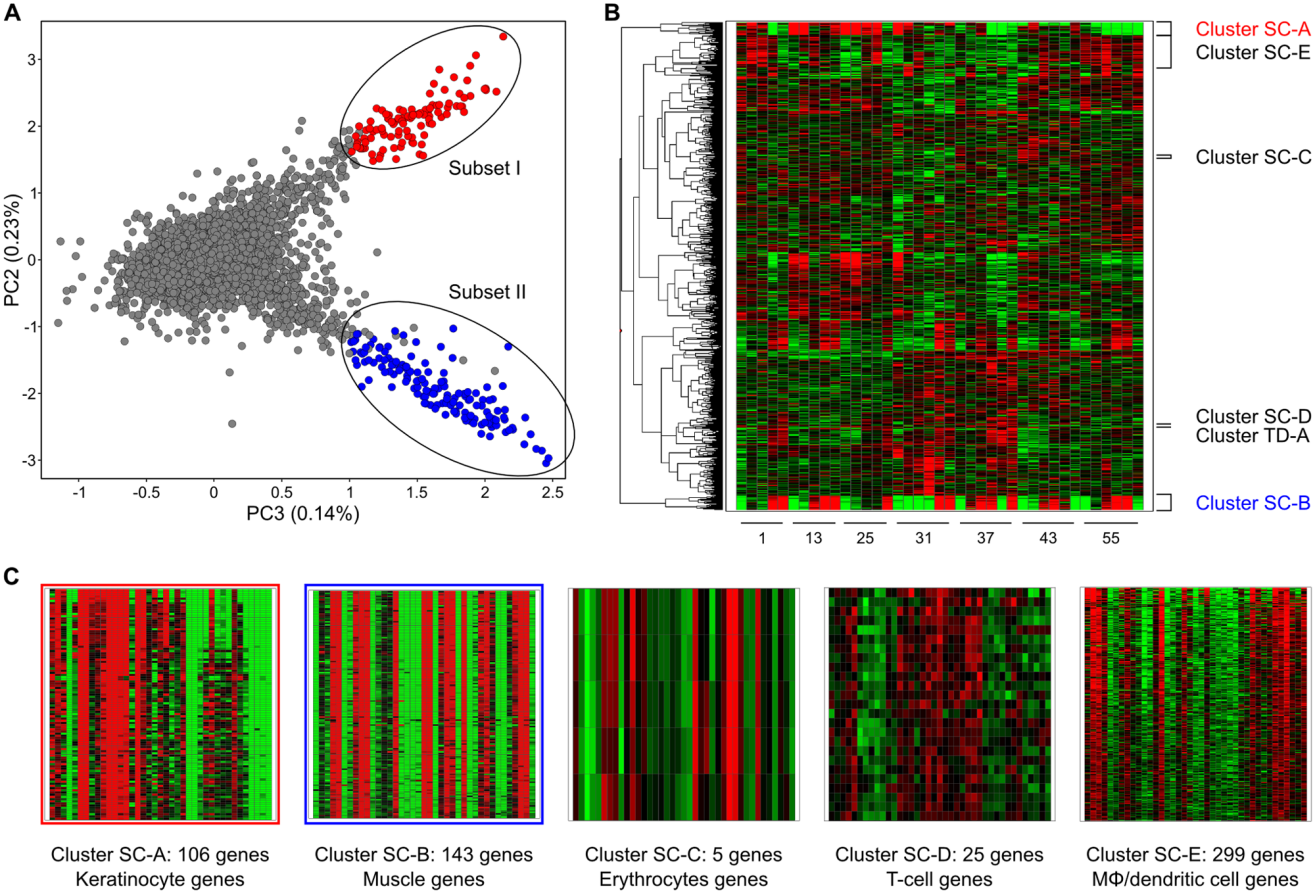
Discovering clusters of genes with very similar gene expression. A: PCA plot of all genes using the untreated WT samples. Indicated are Subsets I and II, with 155 and 109 genes respectively. Red, genes in Subset I that are also in Cluster SC-A. Blue, genes in Subset II that are also in Cluster SC-B; B: A heatmap of microarray signals in all untreated WT samples of the top 5,000 genes with the biggest variance over the whole experiment. The five Sample-Composition and one Time-of-Day specific clusters as explained in the main text are indicated. C: Zoom-in of the five Sample-Composition clusters to reveal the similar expression per cluster over all WT samples.

In search for variation caused by the factor Sample-Composition, we visualized in a PCA plot the genes against the principal components calculated from their expression levels in the untreated WT samples. This revealed at least three major subpopulations (Fig 2A) of genes that behave similarly and we selected two subsets (I: 109 genes and II: 155 genes), with apparent divergent gene expression. To evaluate these subsets, we selected the top 5,000 genes that were most variable over all samples as we felt they represented the most relevant biological changes in the data. We clustered these genes in a heatmap (Fig 2B) and mapped the observed PCA subsets I and II to it. Almost all subset genes were found back in the associated Clusters Sample-Composition (SC)-A (106 genes) and SC-B (143 genes) in the heatmap (Fig 2B and 2C and S3 Table).

**Fig 3:**
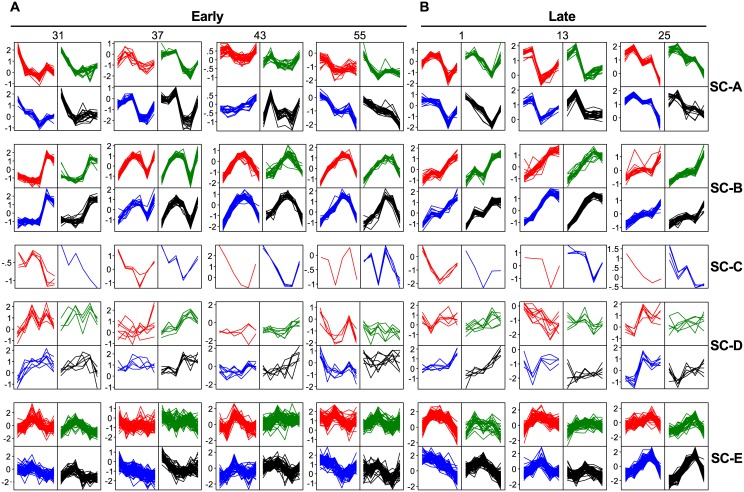
Self-organizing maps of gene clusters with similar gene expression. 2x2 Self-Organizing Maps (SOMS) per individual, untreated WT mouse (#) of the scaled gene-expression profiles of the gene clusters from the heatmap of Fig 2 (S3 Table) for both the Early (0, 1, 2, 3, 4, 5 hours) and Late (0, 7.5, 9, 10.5, 12 hours) time points.

To analyze the expression behavior of these gene clusters in individual mice, we sorted the gene profiles using Self-Organizing Maps (SOMS) (Fig 3, Clusters SC-A and SC-B) for each cluster and each mouse individually. From this SOMS analysis, it immediately became obvious that all genes in a cluster have, *within mice*, highly similar RNA-level profiles over time. However, between mice, the same clusters of genes show quite different RNA-level profiles. This combination of an almost identical RNA-level profile of a large number of genes within a mouse, combined with different profiles between mice, plus a time-independent profile, is a telltale for differences in sample composition. Formally, we cannot distinguish the mingled factors “Sample-Composition” and “Time-of-Day”. Yet, the huge differences (up to 32x) in RNA level between samples taken one hour apart from one mouse, plus the completely different profiles from mice sampled at almost identical time points, does suggest that Sample-Composition might be the most prominent confounding factor here.

If these clusters are primarily caused by sample composition variation and the genes involved are most likely cell type-specific genes, then it should be possible to identify the cell types that vary across the different samples. To this end, we started by simply looking at gene symbols or gene descriptions. As expected, there were many similar genes present in each cluster (S3 Table). For Cluster SC-A this was most striking in that ~85% of the annotated genes were either Keratin (*Krt*) or Keratin associated protein (*Krtap*) genes. With so many Keratin (associated) genes, it is most likely that this whole cluster represents Keratinocyte-specific genes. More importantly, it means that there are severe differences in the number of keratinocytes in each sample. This may be caused by differences in the thickness of the epidermis, which can be a result of many factors.

For Cluster SC-B, it is less obvious from the gene descriptions what underlying cell type is, although this cluster contained several genes related to muscle function such as: myosin light chain (*Myl1*), myosin heavy chain (*Myh1* and *4*), and actin (*Acta1*) (S3 Table). We therefore argued that if genes of a cluster represent a specific cell type, then these genes should show co-expression in a gene-expression tissue atlas. For this we turned to BioGPS, a well-known gene-expression tissue atlas, and looked for the tissue-expression profile of a typical gene from Cluster SC-B: *Myl1*. The associated tissue-expression profile (S3 Fig) immediately made it obvious that this gene cluster may be caused by differential contributions of muscle cells. This is supported by the finding that, of all the genes present in Cluster SC-B, 41% showed a tissue gene-expression profile that in BioGPS strongly correlated (≥0.85) with that of *Myl1* (S4 Table).

These are examples of variability in sample composition caused by differential sampling of static cells. Another potential reason for such variability is influx or outflow of mobile cells to the sampling area of the skin [40,41]. To test this, we reversed the previous argumentation: if we look at cell type-specific genes in mobile–blood derived–cells, such as erythrocytes or immune cells, then we should be able to find clusters of genes that are strictly co-expressed with known cell type-specific genes. When we searched for the erythrocyte-specific gene Hemoglobin alpha 1 gene (*Hba-a1*) in the heatmap (Fig 2B and 2C), we did find it back in a small cluster (Cluster SC-C, S3 Table) together with 4 other erythrocyte specific genes: *Hba-a2*, *Hbb-b1*, *Hbb-b2*, and *Alas2*. These genes are strictly co-expressed as expected (Fig 3), whilst showing a unique gene-expression profile for each mouse. Although we cannot rule out the contribution of the varying amount of blood vessels in the biopsies, it seems clear that the number of erythrocytes, i.e. differential skin blood flow affected the RNA levels during the experiment. This is not hard to imagine as temperature, stress, but also pressure on the skin during biopsy can affect the skin blood flow.

A similar result was achieved by looking at the T-cell specific CD3 genes: *CD3d*, *CD3e*, and *CD3g*. A small cluster (Cluster SC-D, Fig 2B and 2C) of 25 genes was found with several other T-cell specific genes, such as: *Cd7*, *Prf1*, *Trat1*, *Cd94*, and *Nkg7* (S3 Table). Again, their expression was quite similar within one mouse, but distinct between mice (Fig 3). It is obvious that the observed gene-expression is a combination of gene-expression regulation and number of cells in a specific sample. Immune cells are quite mobile [41,42], have a high RNA load, and are often actively recruited to specific locations in the body of an organism [41,42]. This might explain why differential expression of immune-related genes is often found in *in-vivo* transcriptomics experiments (e.g. [43]).

As a final example for the factor Sample-Composition, we looked at macrophages and dendritic cells. For this, we took a naïve approach by looking for these names in the gene description (S3 Table). We found 90 genes with either name in their description and we selected a cluster (Cluster SC-E) of 299 genes in the heatmap (Fig 2B and 2C) in which 50 of those genes were present. Although the similarity of the gene expression profiles within one mouse was less absolute, the differences between mice were still very profound (Fig 3). Macrophages and dendritic cells can migrate [41,42], but are also present in different numbers in the skin of a mouse [41,42]. As such this group represents a group type in between static and mobile cells.

Altogether, given these results, it is very likely that the observed differences in gene expression between the samples are caused primarily by differences in cell composition of the sample as no coherent profiles across mice was observed. So these genes suffer profoundly from this confounding factor. However, it is likely to be affecting many more genes than we have shown here. First of all, we only took some obvious and predictable cell types. Undoubtedly, many other cell types will succumb to the same faith. Even worse, we did not look at all the genes from each cell type: we only looked at the cell type-specific ones, as those have a similar pattern over all the samples and thus cluster together in a heatmap. However, it is clear that all genes that are expressed in cells that have a variable distribution in the samples will suffer similarly from this confounding factor. The reason why we do not observe them lies in the fact that those genes are also expressed in other cell types, with yet another variable distribution in the samples. The combined differences will result in unrecognizable differences per gene as each cell type will have a different contribution to the total sum of gene expression in each gene. This however also means that these genes cannot be analyzed reliably using this experimental approach.

### Potential confounding factor: Time-of-Day

**Fig 4:**
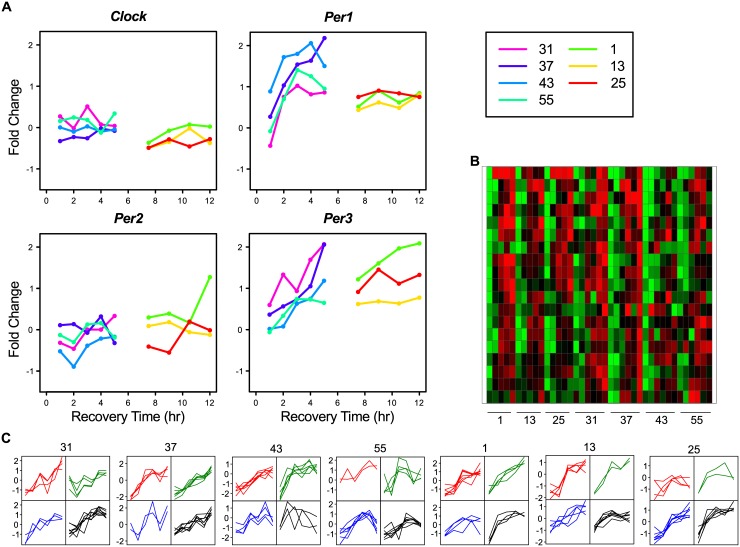
Examples of time-related gene expression from Cluster TD-A. A: Examples of expression profiles over time of genes involved in circadian rhythm for untreated WT samples. B: A heatmap for the 19 genes of Cluster Time-of-Day (TD)-A. C: 2x2 SOMS of the scaled gene-expression profiles per mouse of gene Cluster TD-A.

The factor Time-of-Day obviously relates to circadian rhythm. Core clock components are defined as genes whose protein products are necessary for the generation and regulation of circadian rhythms within individual cells throughout the organism [44]. There are several genes and proteins involved in the complex regulation of the circadian rhythm, which has at least three primary feedback loops. Genes in the positive and negative parts of these feedback loops are: Clock, *Bmal1* (= *Arnt*), *Per1*, *Per2*, *Per3*, *Cry1*, *Cry2*, *Nr1d1* (= *Rev-erbα*), *Nr1d2* (= *Rev-erbβ*), *Rora* (= *Rorα*), *Csnk1e* (= *CK1ε*), *Csnk1d* (= *CK1δ*). The interaction between these genes is described in [44]. To exemplify the effect of Time-of-Day, we looked at the gene-expression profiles of the *Per* genes in untreated WT samples over time (Fig 4A). Of the three *Per* genes, *Per1* and *Per3* were present in the 5,000 most variable genes. Using the same approach as before (Fig 2B), we used these genes to identify a Time-of-Day cluster (Cluster TD-A) of 19 genes that had similar expression over all samples (Fig 4B and S5 Table). In this cluster also other genes known to be involved in circadian rhythm were present such as; *Dbp*, *Nr1d2*, *Tsc22d3*, *Zbtb16*, and *Tef*. As can be observed in the SOMS of this cluster (Fig 4C), all genes have a clear time dependent gene-expression profile. This is quite similar within one mouse, but between mice major differences are still present. In analogy with the genes affected by the factor Sample-Composition, the genes in this cluster cannot be used in these type of studies because Time-of-Day is an evident nuisance factor.

It is obvious that the 19 genes in Cluster TD-A are not the only circadian-rhythm involved genes and that those unknown genes are as such also affected by the factor Time-of-Day. Identifying them is not a simple assignment, as all mice might have different timing of their circadian rhythm. We used the fact that i: this phenomenon is already present in all untreated mice, and ii: the involved genes should display a more gradual change i.e. low variation, than genes affected by the previous factor Sample-Composition. Translating the first criterion, we selected genes that have in Untreated WT samples a Fold Change (FC) ≥ 2 expression difference between at least two time points in each mouse. This resulted in 528 genes in the Untreated WT Early samples and 547 in the Untreated WT Late samples. That these genes are likely involved in circadian-sensitive processes can be read from the fact that they contained 68% and 47% respectively of the genes from Time-of-Day Cluster TD-A. As to the second criterion, we ranked the genes based on variation (S5 Table). The Cluster TD-A genes ranked here with the genes having the lowest variation. Hence, we selected the first 277 Early and 93 Late genes, which resulted in a union of 333 genes (Group TD-B) for the factor Time-of-Day.

**Fig 5:**
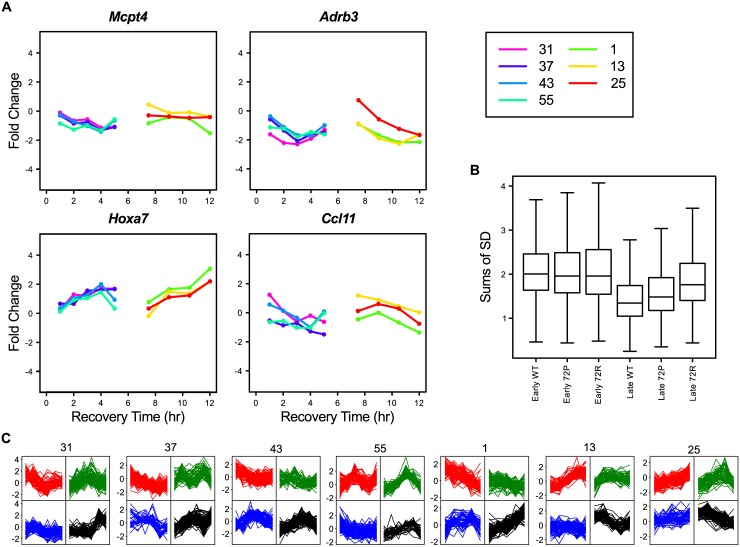
Examples of time-related gene expression from Group TD-B. A: Examples of time-related expression profiles for untreated WT samples for genes from Group TD-B. B: A boxplot for the variation of the 333 genes in Group TD-B. C: 2x2 SOMS per mouse of the scaled gene-expression profiles of gene group TD-B.

The genes in group TD-B showed a more diverse profile picture (Fig 5A and 5C) as compared to the Sample-Composition gene clusters, indicating that these genes, although all related to time-of-day, have different expression profiles over time. Also, it was clear that the TD-B genes had a much higher variation at the Early time points then at the Late time points (Fig 5B), which forms additional evidence for a time-of day effect in these genes. Obviously, gene expression of many more genes is affected by the circadian rhythms of the animal and/or the cells. For instance, over 3,000 transcripts are under circadian control in the liver [45]. This is supported by the fact that not all of the true circadian rhythm regulated genes of Custer TD-A are present in Group TD-B. The explanation for not detecting them might be found in our naive approach with a relative high FC cut-off of 2, as well as lack of true replicates.

### Potential confounding factors: Handling- & Biopsy-Stress

Handling is the entire repertoire of manipulations that the mice undergo during the whole experiment and taking a biopsy is a part of this. As such, we will consider all handling here as one confounding factor. One group of genes that could be involved in handling are wound-healing genes. There are several genes known to be involved in wound-healing: *Fos*, *FosB*, *Mkp-1*, *Cd14*, *Ccl9*, *Pigf*, and *Mcp-5* [46]. However, the expression of these genes does not cluster together in our heatmap (Fig 2B). Hence, we could not define a cluster based on known wound-healing genes.

**Fig 6:**
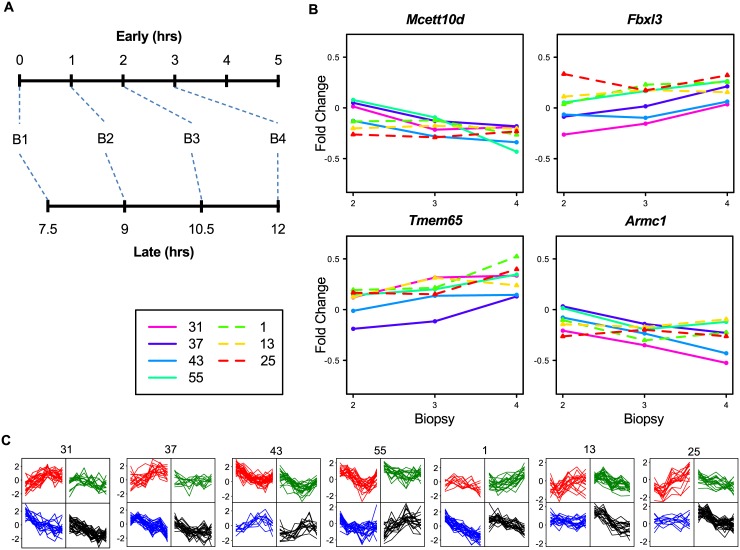
Examples of biopsy-stress-related gene expression from Group HS-A. A: Re-labeling of samples from time points to biopsy-order number (B#, cf. main text). B: Examples of Handling-Stress expression profiles for untreated WT samples for genes from Group HS-A. C: 2x2 SOMS per mouse of the scaled gene-expression profiles of the 83 genes in group HS-A.

Another way to identify genes that are affected by this factor in this experiment, is to evaluate the two untreated WT time series by using the biopsy sampling number instead of the recovery time. For this, we assumed that the biopsy sampling sequence in the late series restarts at recovery time point 7.5 hours, because we assume the mice have recovered by then from their biopsy at time point zero. So, we re-labeled the early recovery time points: t0, t1, t2, t3, t4, t5 into biopsy points: b1, b2, b3, b4, b5, and b6, respectively and the late recovery time points: t0, t7.5, t9, t10.5, t12 into biopsy points: b1, b2, b3, and b4 (Fig 6A). This allowed us to identify DEGs between these biopsy points, which resulted in 2,585 DEGs. In order not to overestimate the genes related to factor Handling-Stress we chose the 83 DEGs (Group HS-A) with a SD<0.32 over all untreated WT samples (S6 Table) as genes affected by handling stress, which left behind most SC-A, SC-B and TD-A genes that were found ranking between the DEGs with higher variation. The HS-A genes have low variation as well as low fold change when plotted against the biopsy points (Fig 6B) and quite some variation when plotted against recovery time points (Fig 6C). This supports our conclusion that these genes genuinely represent the handling stress response.

### Potential confounding factor: Individual-Mouse

Then there are still other unknown (genetic) factors that can lead to differences between individual mice. These effects can be identified and quantified by analyzing whether the expression of a gene at all sample points from one mouse differs substantially from those in the other mice. To examine this, we estimated the maximal Individual-Mouse effect for each gene over all untreated WT mice in the ANOVA model of our experiment and identified 628 genes by applying an arbitrarily-chosen threshold of coefficient value ≥ 0.5. There is a substantial overlap with the previously identified clusters with for instance all SC-A genes and about half of the TD-B genes. We defined a group of 391 genes for the factor Individual-Mouse (group IM-A) that have no overlap with any other group or cluster.

**Fig 7:**
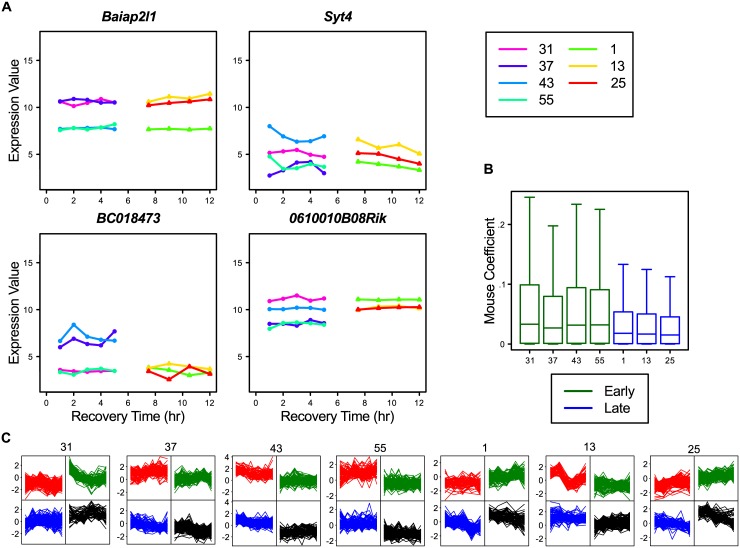
Examples of individual-mouse-related gene expression from Group IM-A. A: Examples of expression profiles for untreated WT samples for genes from Group IM-A. B: Boxplot of absolute ANOVA Individual-Mouse effect of all genes per untreated WT mouse. C: 2x2 SOMS per mouse of the scaled gene-expression profiles of all 391 genes in group IM-A.

The gene-expression profiles of the IM-A genes showed the expected differences between mice (Fig 7C), where there seemed to be genes with two or three distinct expression levels (Fig 7A). As 99% of these genes also are present in the top 5,000 variable genes, we looked for obvious sub-clusters in the heatmap. The IM-A genes clustered quite prominently in several clusters (results not shown), meaning that the observed factor Individual-Mouse is a result of several broad individual-mouse effects. Besides these gene-clustered individual-mouse effects, it was also noticeable that the average ANOVA Individual-Mouse effect for the Late mice was different from those used at the Early mice, which suggests there might be an interaction between the two factors: Time-of-Day and Individual Mouse (Fig 7B). Given the substantial Individual-Mouse effects (maximum of log2(2)) we observed, the factor Individual-Mouse has to be seriously considered during gene-expression analysis. This is underlined by the fact that, when we determine DEGs for untreated or UVR exposed WT mice, there is a substantial difference in DEG numbers if we compare the ANOVA analysis with and without Individual-Mouse in the model (Table 3). Hence, in this experiment, Individual-Mouse is a prominent confounding factor.

**Table 3:** Differentially-expressed genes (DEGs) in untreated and treated WT mouse.

		DEGs in untreated WT samples		DEGs in treated WT samples	
Contrast		UV-Dose & Recovery-Time effect	UV-Dose & Recovery-Time + Individual-Mouse effect	UV-Dose & Recovery-Time effect	UV-Dose & Recovery-Time + Individual-Mouse effect
t0 vs	t1	0	0	68	161
	t2	0	30	2,226	3,200
	t3	86	293	5,300	6,466
	t4	52	264	7,342	8,486
	t5	28	68	4,395	5,649
t0 vs	t7.5	0	1	0	2
	t9	1	3	6	11
	t10.5	4	16	196	394
	t12	10	47	243	505

### The effects of confounding factors on transcriptome analysis

In the previous paragraphs we have characterized several potential confounding factors and the genes which expression is clearly affected by it. Now we will turn our attention to the effects these factors might have on the analysis and interpretation of the transcriptome response in our experiment. Usually, transcriptome analysis starts by identifying DEGs in relevant contrasts using an ANOVA-based analysis. Here, the contrasts are from each recovery time point to either the associated time point zero or the identical untreated recovery time point (Tables 3 and 4).

**Table 4:** Comparing confounding factors derived clusters, groups and differentially-expressed genes (DEGs).

				Confounding factor				
				Sample-Composition	Time-of-Day	Handling-Stress	Individual Mouse	All
				SC-A, B, C, D and E	TD-B	HS-A	IM-A	
			DEGs	578	333	83	391	1,343
UV-B	Contrast		#	% Overlap	% Overlap	% Overlap	% Overlap	% Overlap
0	t0	t1	0	-	-	-	-	-
		t2	30	3	57	0	3	63 ^a^
		t3	293	11	22	1	3	37 ^a^
		t4	264	6	22	1	5	34 ^a^
		t5	68	3	41	1	3	49 ^a^
	t0	t7.5	1	0	100	0	0	100
		t9.0	3	0	67	0	0	67
		t10.5	16	0	63	0	0	63 ^a^
		t12.0	47	13	45	2	4	64 ^a^
High	t0	t1	161	1	6	0	4	11 ^a^
		t2	3,200	3	3	1	2	8 ^a^
		t3	6,466	3	3	1	2	9 ^a^
		t4	8,486	3	2	1	2	8 ^a^
		t5	5,649	3	2	1	2	8 ^a^
Low	t0	t7.5	2	-	-	-	-	-
		t9.0	11	18	55	0	0	73 ^a^
		t10.5	394	24	13	1	2	40 ^a^
		t12.0	505	21	12	1	2	36 ^a^
High	t0_u	t0_t	0	-	-	-	-	-
	t1_u	t1_t	5	-	-	-	-	-
	t2_u	t2_t	501	0	1	1	2	3
	t3_u	t3_t	1,149	0	0	1	1	2
	t4_u	t4_t	3,185	0	1	1	1	2
	t5_u	t5_t	1,206	0	0	1	1	2
Low	t0_u	t0_t	0	-	-	-	-	-
	t7.5_u	t7.5_t	0	-	-	-	-	-
	t9.0_u	t9.0_t	0	-	-	-	-	-
	t10.5_u	t10.5_t	1	-	-	-	-	-
	t12.0_u	t12.0_t	2	50	0	0	0	50

^a^ these values were found significant (Pval<0,05) in the over-representation analysis

**Fig 8:**
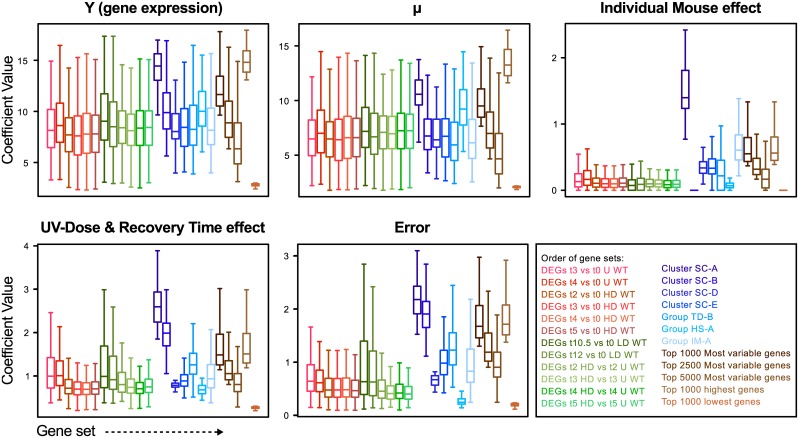
The contribution of the various ANOVA components. The ANOVA components: Y = μ + Individual Mouse effect + UV-Dose & Recovery-Time effect + error evaluated in boxplots for the indicated groups and clusters of genes.

All these results, with respect to the potential confounding factors and DEGs, also prompted us to take a renewed look at the ANOVA model we applied to analyze our experiment. For this we plotted the different ANOVA elements for various gene clusters and groups (Fig 8). As can been seen, the gene clusters and groups behave logically, such as Cluster SC-A (associated with keratinocytes) showed an extreme Individual-Mouse effect. This means that individual mice probably differ quite substantially in their number of keratinocytes due to different epidermis thickness. The extreme low Individual-Mouse effect of Cluster SC-B (associated with muscle cells) genes, divulges that this cluster is caused by sampling effects. For clusters SC-D, -E and group TD-B also relatively high Individual-Mouse effects are found, indicating that these clusters harbor a substantial Individual-Mouse effect. Finally, the Individual-Mouse group has an expected relative high Individual-Mouse effect. Observing the ANOVA element UV-Dose & Recovery-Time effect shows that the DEGs have a relatively modest average effect as compared to that of gene clusters and groups. Although ANOVA performs as it is supposed to, by selecting genes with small but consistent differences, one could argue what the significance is of these DEGs given the huge differences that are observed in the expression of many other non-DEG genes.

The ANOVA analysis resulted in several hundred DEGs in the untreated WT contrasts (union = 437 genes) and several thousand DEGs (union = 11.891 genes) in the treated WT contrasts (Tables 3 and 4 and S7 Table). There is an obvious difference in Low dose as compared to High dose, but altogether it means that almost 50% of all inquired transcripts show a differential gene expression somewhere in the experiment. This makes it unlikely that the DEGs represent a specific UV → DNA damage → DNA repair response.

When we evaluated the DEGs, several points surfaced: The contrasts that were based on time matched controls, showed, although still over 3,000 at one point, substantially less DEGs as those to their own time point zero. This is to be expected since time matched points are obviously less different. However, even in these contrasts the numbers of DEGs seem to change in a time dependent fashion, which are most likely explained by Inter-individual differences in genetic and epigenetic control of transcription and protein synthesis and feedback loops. All of the 437 DEGs found in the untreated WT contrasts were also identified as WT-treated DEGs (Table 4, S8 Table, and S4 Fig) and could be considered false-positives with respect to the UVR exposure.

To evaluate the observed DEGs even further, we identified those that had an overlap with the top 5,000 most variable genes from our heatmap (S7 Table). This overlap was high in the untreated and low-dose samples, but rather low in the high-dose treatment. The DEGs were also checked for overlap with the gene clusters and groups. Depending on the cluster or group the overlap ranged from 0% (Cluster SC-A) to high percentages (Group TD-B). Adding up the percentages overlap, it turned out that over 34% of DEGs from the untreated contrasts could be explained by their presence in the clusters or groups associated with the analyzed confounding factors. For DEGs from the treated contrasts, this was still 8% and up. Also the over-representation analysis shows that most contasts have significant numbers of genes from the clusters/groups in the DEGs. Collectively this means that many ANOVA-identified DEGs actually may be a result of bias due to confounding factors, rather than a response to UVR exposure.

### Concluding remarks

We set out to identify uncontrollable confounding factors that might confuse transcriptome analysis of *in-vivo* experiments. Several of those confounding factors were found and their impact on the experiment data seems severe. One has to bear in mind that our unique experimental set-up allowed a thorough exploration of the confounding factors, whereas most *in-vivo* studies this cannot be done by lack of multiple samples from one individual. Still we feel that in many *in-vivo* studies identical or similar confounding factors play an important role and even though being undetected, severely influence the results.

The most prominent confounding factor we observed was Sample-Composition, caused by mouse-dependent skin-composition differences, sampling variation and influx/efflux of mobile cells. We illustrated the effects by using cell type specifically expressed genes as these provide the clearest effects. However, it is apparent that these variations in sample composition also affect the cumulative expression levels of other cell non-specific genes. Hence, variations in sample composition will have an effect on the measured expression of many genes, but since it is an accumulation of contributions of many cell types, they cannot be recognized, nor fixed. Especially in mobile cells combined influx and activation will make it impossible to interpret these observed gene-expression differences that mimic transcription induction.

From the effects of the confounding factors Time-of-Day and Handling Stress, it became evident that even the most controlled experiments will suffer from these effects. As these effects are unavoidable, the affected genes will be hard, if not impossible, to study *in-vivo*.

We were somewhat surprised by the sizable differences at the gene-level between individual mice. In this experiment, many genes seem to be affected by this confounding factor and it also played a role in other confounding factors. The observed differences were so substantial that we have to conclude that range-finding using single mouse samples, as we have advocated earlier [30], is not a good approach for this experimental setting. Moreover, in our experiment set-up with several samples per mouse, we were able to model the individual-mouse effect in our ANOVA analysis. As most *in-vivo* experiments are based on single samples per mouse, this is impossible; hence these huge individual-mouse effects go uncorrected.

Obviously ANOVA can be a good method to deal with the effects of noise as introduced by confounding factors [27–29]. However, we feel that one should wonder what the biological meaning is of the small differences in gene expression of many ANOVA-selected DEGs as other “noisy” genes in these cells exhibit huge differences. In these cases it seems that the common “one-gene-at-the-time” ANOVA analysis is focused so much on reproducible differences, that it ignores all other expression differences that go on in a cell. From the combined observations in this study, we feel that cells may display something like a “gene-transcription status”, in which gene expression of many genes moves in a network fashion. Proper analysis would require a systems biology approach with appropriate modeling and analysis of groups of genes, rather than individual ones.

Our overall conclusion is that *in-vivo* experiments are extremely prone to confounding factors. The major impact the confounding factors have on the outcome makes that the set-up, analysis, and interpretation of such experiments should be undertaken with the utmost prudence.

## Supporting Information

S1 Fig Mice sex-determination check via gene expression of *Xist*. The expression of female-specific gene *Xist* plotted for each of the replicated five or six sample set per mouse. Mouse 42 and 51 are female as all samples taken from them show *Xist* expression. Trp53-Genotypes are colored: Green, WT; Blue, *Trp53*-72P mutant; Red, *Trp53*-72R mutant.
(PDF)

S2 Fig RNA yields. Comparison of the rRNA (via total-RNA) and mRNA (via aRNA) yields. A, averaged total-RNA yields over four mice for the untreated, high-dose, and low-dose samples with error bars of SD; B averaged aRNA yields over four mice for untreated, the high-dose, and low-dose samples with error bars of SD. C, individual total-RNA yields over 4 mice for the untreated and treated high-dose and low-dose samples; D, individual aRNA yields over 4 mice for the untreated and treated high-dose and low-dose samples.
(PDF)

S3 Fig Examples of tissue-specific gene expression from Cluster SC-B. BioGPS images showing the tissue-specific expression of genes *Myl1* and *Adssl1*, present in Cluster SC-B.
(PDF)

S4 Fig Overlapping DEGs. VennDiagram showing the overlap between the DEGs found in untreated WT, High Dose (HD) and Low Dose (LD) (S7 Table).
(PDF)

S1 Table Complete experiment set-up. (XLSX)

S2 Table RNA yields. (XLSX)

S3 Table Top-5000 heatmap. (XLSX)

S4 Table BioGSP output. Genes with tissue specific expression highly correlation (≥0.85) with that of *Myl1*.
(XLSX)

S5 Table Variety ranked genes Early and Late. (XLSX)

S6 Table Handling Stress DEGs ranked on variety. (XLSX)

S7 Table Comparing confounding factors derived clusters, groups and differentially-expressed genes (DEGs), complete table. (XLSX)

S8 Table Differentially-expressed Tpr53-responsive genes. (XLSX)

S1 Text Data quality control. (DOCX)
