# Supplementary figures and images for: Confounding Factors in the Transcriptome Analysis of an In-Vivo Exposure Experiment
Source: PLoS One. 2016 Jan 20;11(1):e0145252. doi: 10.1371/journal.pone.0145252 (PMC4720430; doi:10.1371/journal.pone.0145252)

# *Xist*

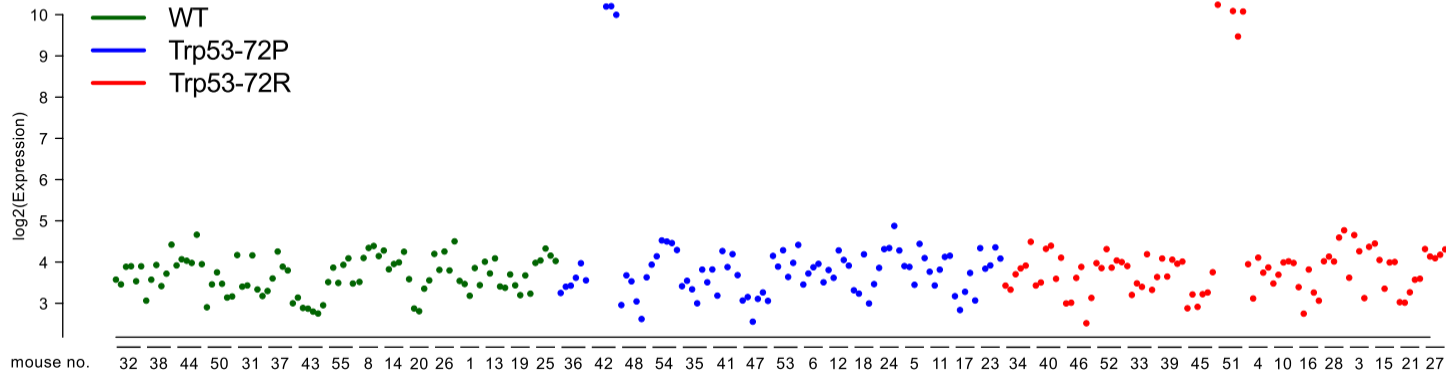

Supplement: S1 Fig — The expression of female-specific gene Xist plotted for each of the replicated five or six sample set per mouse. Mouse 42 and 51 are female as all samples taken from them show Xist expression. Trp53-Genotypes are colored: Green, WT; Blue, Trp53-72P mutant; Red, Trp53-72R mutant. (PDF) [file pone.0145252.s001.pdf]

A

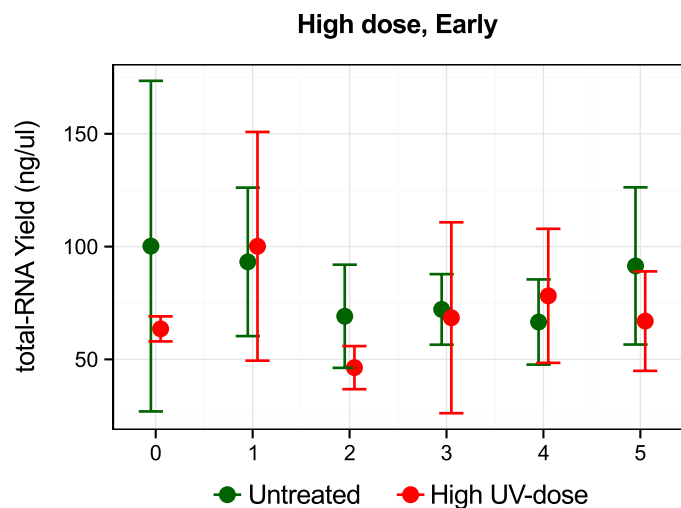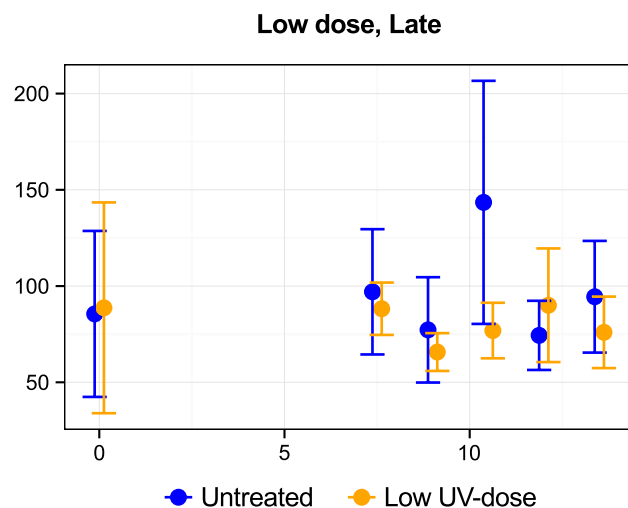

B

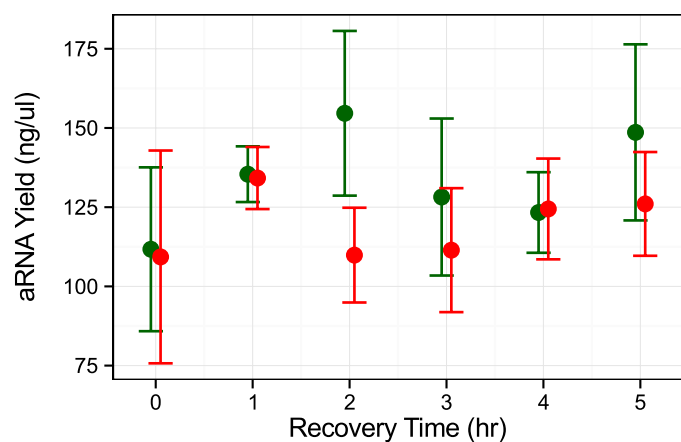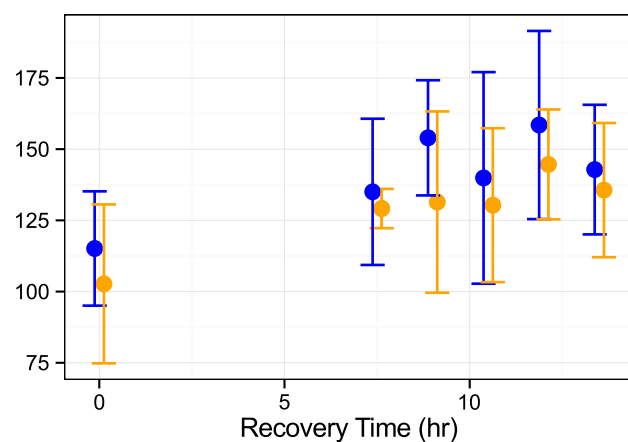

C

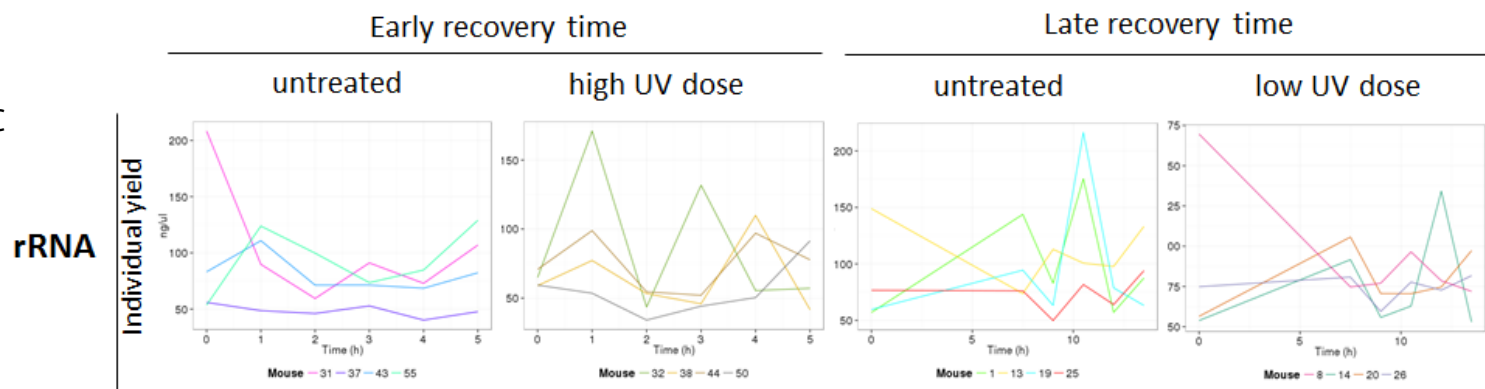

D

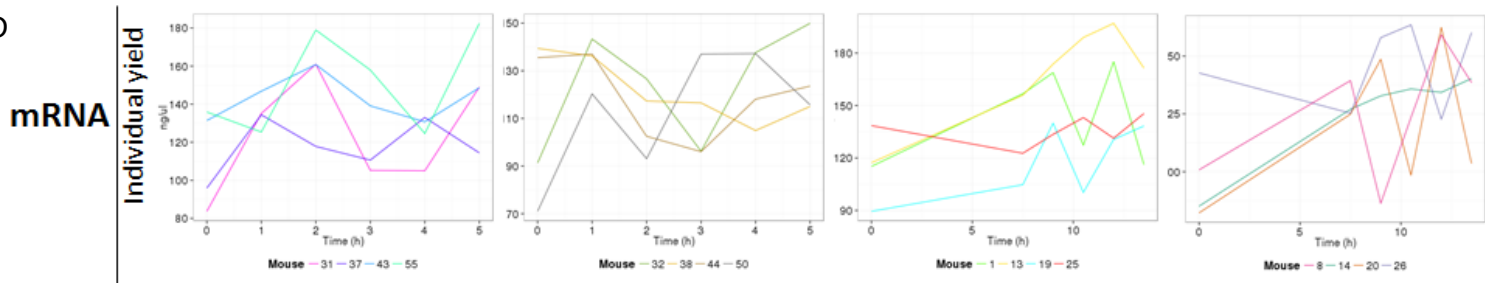

Supplement: S2 Fig — Comparison of the rRNA (via total-RNA) and mRNA (via aRNA) yields. A, averaged total-RNA yields over four mice for the untreated, high-dose, and low-dose samples with error bars of SD; B averaged aRNA yields over four mice for untreated, the high-dose, and low-dose samples with error bars of SD. C, individual total-RNA yields over 4 mice for the untreated and treated high-dose and low-dose samples; D, individual aRNA yields over 4 mice for the untreated and treated high-dose and low-dose samples. (PDF) [file pone.0145252.s002.pdf]

**Myl1**

**Adss1l**

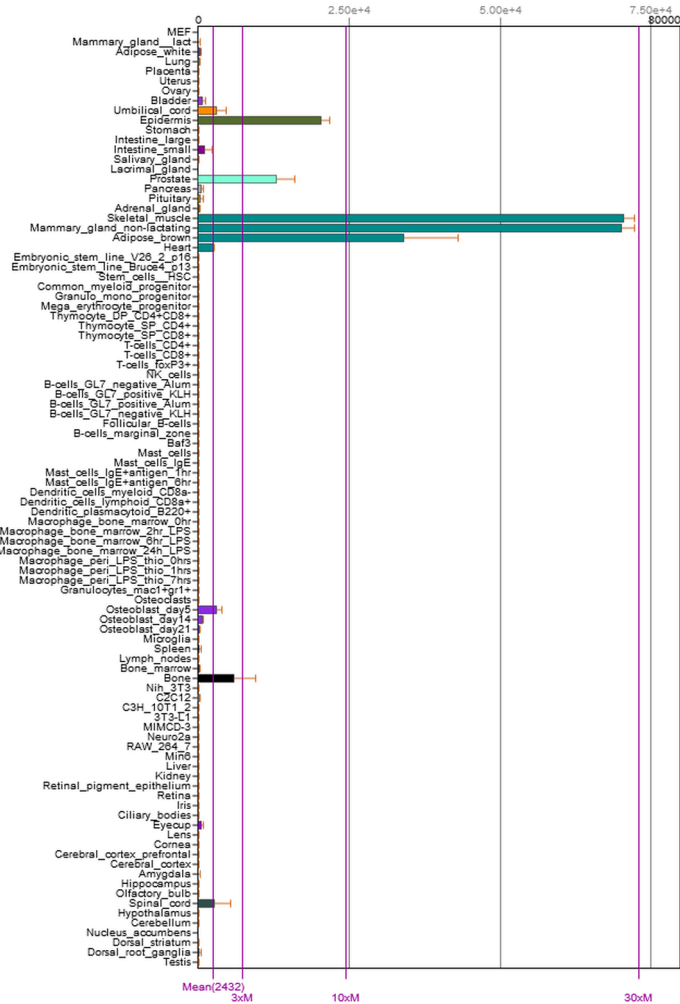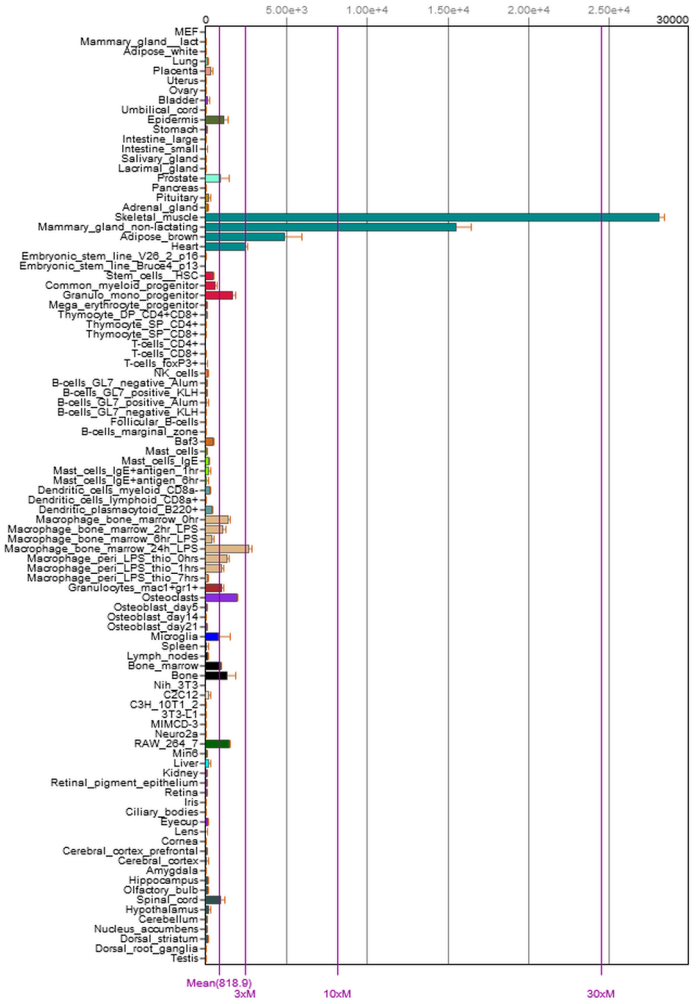

Supplement: S3 Fig — BioGPS images showing the tissue-specific expression of genes Myl1 and Adssl1, present in Cluster SC-B. (PDF) [file pone.0145252.s003.pdf]

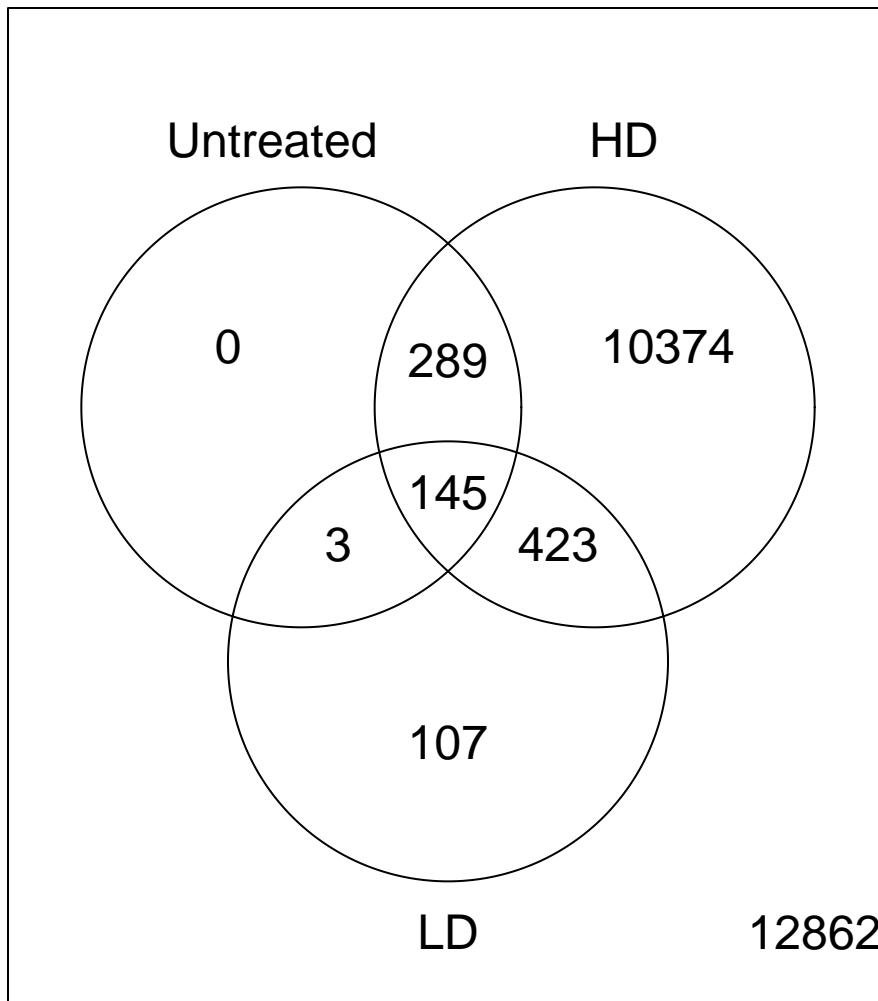

Supplement: S4 Fig — VennDiagram showing the overlap between the DEGs found in untreated WT, High Dose (HD) and Low Dose (LD) (S7 Table). (PDF) [file pone.0145252.s004.pdf]
