# Supplementary material for: Confounding Factors in the Transcriptome Analysis of an In-Vivo Exposure Experiment
Source: PLoS One. 2016 Jan 20;11(1):e0145252. doi: 10.1371/journal.pone.0145252 (PMC4720430; doi:10.1371/journal.pone.0145252)
Supplement: S1 Text — (DOCX) [file pone.0145252.s013.docx]

## Data quality control

Correct information about the samples in the experiment is of the utmost importance, therefore, after the last biopsy, we confirmed the *Trp53* genotype of each mouse by a PCR check using the DNA from a tail section (data not shown). The sex of the mice in the experiment was checked by evaluating expression of *Xist*, a gene-expression marker for female cells (S1 Fig). All samples taken from mice 42 and 51 clearly showed *Xist* expression and these samples were removed from the experiment (S1 Table), as gender-specific differences would introduce extra variation undesired for our specific research question. Furthermore, during the analysis process it became clear that one sample (untreated WT t0) was contaminated by liver cells. This was obvious from the unique presence of RNA from many liver-specific genes (results not shown). It turned out that this was also the only mouse that showed bite marks during the experiment; hence all samples from this mouse were also removed from the study (Table 1).

Based on earlier experiences [1], we examined the rRNA and mRNA yields to see if there were differences that correlated with one of the experimental parameters. First we looked at the total-RNA yield of all samples (S2A and S2C Figs), which represents the rRNA yield. Although we observed substantial differences in rRNA yields, ranging from 33 ng/μl to 354 ng/μl (S2 Table), there seemed no obvious correlation between rRNA yield and any experimental variable (S2C Fig). To estimate the relative mRNA yields, we looked at the *in-vitro* amplified aRNA of all samples (S2B and S2D Figs), which ranged from 54 ng/μl to 197 ng/μl (S2 Table). Similar to the rRNA yields, no obvious correlation between mRNA yield and any experimental variable was observed (S2 Fig). Even between samples taken from one mouse there was no consistency with respect to RNA yields.

1. Bruning O, Yuan X, Rodenburg W, Bruins W, van Oostrom CT, Rauwerda H, et al. Serious complications in gene-expression studies with stress perturbation: An example of UV-exposed p53-mutant mouse embryonic fibroblasts. Transcription. 2010;1: 159–164. doi:10.4161/trns.1.3.13487
